# Analysis of Some Risk Factors of Active Tuberculosis in Three South Florida Counties

**DOI:** 10.7759/cureus.19852

**Published:** 2021-11-24

**Authors:** Milhenka Auguste, Christine McGuire-Wolfe, Alina Alonso, Okelue E Okobi

**Affiliations:** 1 Family Medicine, Lakeside Medical Center, Belle Glade, USA; 2 Infectious Disease, Florida Department of Health in Palm Beach County, Palm Beach, USA; 3 Infectious Disease, University of South Florida College of Public Health, Tampa, USA; 4 Epidemiology and Public Health, University of South Florida College of Public Health, Tampa, USA; 5 Public Health, Florida Department of Health in Palm Beach County, Palm Beach, USA

**Keywords:** tuberculosis (tb), diabetes mellitus, hiv/aids, race, prevalence study, latent tuberculosis treatment

## Abstract

Background

With tuberculosis (TB) being among the top 13 leading cause of death and second leading infectious disease killer next to COVID-19 globally, there is a need for continued study and a better understanding of the risk factors and management approaches. One in five tuberculosis (TB) deaths occurs in individuals who have contracted human immunodeficiency virus (HIV). However, other risk factors play a role in its morbidity pattern. Therefore, descriptions of these comorbidities between TB, HIV, and other risk factors such as diabetes are needed.

Method

A retrospective, descriptive study was conducted to evaluate the prevalence of TB and its relationship with some risk factors (HIV, diabetes, race, ethnicity, end-stage renal disease, post-organ transplant, recent contact with active TB, and other non-HIV immunosuppressive conditions) using data from three South Florida counties (Dade, Broward, and Palm Beach) from 2010 to 2019 retrieved from the CDC’s *Report of Verified Case of Tuberculosis* (RVCT).

Results

A total of 2437 cases of TB were reported between 2010 and 2019. There was approximately a 14% positive rate among the three counties for HIV. In contrast, 47% of the individuals with active TB in all three counties were also diagnosed with diabetes mellitus. An average of 25% of the active TB cases in these counties had a concurrent immunosuppressive condition other than HIV. Known contact with another active TB case was an identified risk factor in 18%, 17%, and 29% of reported TB cases in Dade, Broward, and Palm Beach counties, respectively.

Discussion

The HIV status of patients with TB in these three counties was predominantly negative, in contrast to initial theories. The presence of diabetes mellitus was associated with a diagnosis of TB or latent tuberculosis infection (LTBI) within the studied population.

Conclusion

Screening for latent tuberculosis infection (LTBI), compliance, and promotion of LTBI management in newly diagnosed and uncontrolled diabetics may be a successful prevention strategy for this high-risk group*. *

## Introduction

With tuberculosis (TB) being among the top 13 leading causes of death and second leading infectious disease killer next to COVID-19 globally [1], there is a need for continued study and a better understanding of the risk factors and management approaches. Between 2016 and 2019, a report of roughly 12,000 TB cases in the USA was observed, resulting in an incidence rate of 3 cases per 100,000 persons [2]. The risk factors that lead to a diagnosis of TB are crucial in efforts to eradicate this disease. In 2019, 51% (n = 4547) of TB cases in the USA were reported from four states: California, Texas, New York, and Florida [2]. In the same year, a total of 71.4% (n = 6364) of reported TB cases in the USA occurred among non-US-born individuals [2]. To significantly reduce the incidence of TB, it is critical to study at-risk populations and implement one of the recommended strategies for managing TB, which is divided into two parts: treating current cases in places with a high incidence of tuberculosis and providing preventative treatment for latent TB infection (LTBI) in areas with a low incidence of tuberculosis [3].

Several risk factors have been historically associated with tuberculosis (TB). According to the CDC, generally, persons at high risk for developing TB disease fall into two categories: (a) persons who have been recently infected with TB bacteria (close contacts with a person with infectious TB disease; persons who have immigrated from areas of the world with high rates of TB; children less than five years of age who have a positive TB test; groups with high rates of TB transmission, such as homeless persons, injection drug users, and persons with HIV infection; and persons who work or reside with people who are at high risk for TB in facilities or institutions such as hospitals, homeless shelters, correctional facilities, nursing homes, and residential homes for those with HIV) and (b) persons with medical conditions that weaken the immune system (HIV infection, substance abuse, silicosis, diabetes mellitus, severe kidney disease, low body weight, organ transplants, head and neck cancers, medical treatments such as corticosteroids or organ transplant, and specialized treatment for rheumatoid arthritis or Crohn’s disease) [4]. According to the CDC, TB was reported in 1753 African Americans in the USA in 2019, accounting for approximately 20% of cases nationally [4]. One in five tuberculosis (TB) deaths occurs among people living with human immunodeficiency virus (PLWH) [5]. HIV weakens the host’s immune system, making the host susceptible to opportunistic infections [6]. Among various illnesses, TB is one of the leading risk factors and causes of death among people living with HIV (PLWH) worldwide [7]. Awareness of the individual elements of HIV and TB will provide a better understanding of how these diseases coexist from an epidemiological standpoint.

The first domestic case of HIV was recorded in the USA in 1981 [8]. In 2017, the incidence rate of HIV was higher in the metropolitan regions of the southernmost USA compared with that of the northeast, west, and midwestern USA [9]. Worldwide, 36.7 million people were living with HIV by 2016 [8]. Insight into the social determinants of health is key to understanding the root cause of HIV incidence in those with multiple sexual partners, intravenous drug abusers, and immunosuppressed and/or immunocompromised individuals.

Global estimates of TB-related illness and deaths among PLWH decreased in 2017 compared with previous years due, in part, to the early diagnosis of latent tuberculosis infection (LTBI), preventive treatment, and early initiation of antiretroviral therapy [9]. While TB disease remains a worldwide public health challenge, screening, early identification of LTBI, and preventative treatment can reduce the likelihood of the development of active TB disease. In addition to HIV infection, the risk factors that are associated with TB activation include diabetes mellitus (20.7%, n = 1845), contact with a person with infectious TB (8.2%, n = 731), or an immunocompromising condition other than HIV (8.2%, n = 73) [6]. Compared with HIV infection, a history of diabetes may be equally influential to the development of active TB among African Americans. In health disparities, it is important to explore the interaction between HIV and TB infection. Compared with Caucasian adults, African American adults in the USA are 60% more likely to be diagnosed with diabetes by a physician [10], with diabetes previously reported to cause a threefold increase in TB in among diabetic patient population (relative risk: 3.11; 95% CI: 2.27-4.26) [11].

It is known that HIV is a leading risk factor for acquiring TB in a population group. However, in this study, that was not the case. This study analyzed the occurrence of TB in patients with HIV and identified other possible common risk factors (race, recent contact with active TB cases, non-HIV/AIDS immunosuppressive conditions, post-organ transplantation, and diabetes) that promote TB activation in the study sample of three South Florida locations: Dade, Broward, and Palm Beach. This sampled population was randomly selected for this study purpose. Reported cases of TB coexisting with HIV, diabetes, and non-HIV-related immunosuppression in three South Florida communities, Dade, Broward, and Palm Beach counties, will be analyzed for trends related to race and ethnicity.

## Materials and methods

Inclusion criteria

The inclusion criteria included the following: (a) all culture-confirmed cases of tuberculosis in Miami-Dade, Broward, and Palm Beach counties of the state of Florida, USA, that was reported in the CDC’s Report of Verified Case of Tuberculosis (RVCT) between 2010 and 2019, (b) being 18 years old or older, and (c) any gender.

Exclusion criteria

The exclusion criteria included the following: (a) change of TB diagnosis during treatment, (b) transfer to another health unit outside the mentioned counties, and (c) any initial preliminary report based on symptoms alone without laboratory confirmation.

Study design

This is a retrospective, observational, descriptive study based on secondary data. We used the epidemiologic data from three randomly selected counties (Miami-Dade, Broward, and Palm Beach) in Florida from 2010 to 2019. Approval from the Florida Department of Health’s Division of Community Health Promotions Internal Review Board was obtained to use TB surveillance data associated with Miami-Dade, Broward, and Palm Beach counties. Data were retrieved from the CDC’s Report of Verified Case of Tuberculosis (RVCT) by the surveillance manager at the Palm Beach County Department of Health.

Definitions

Tuberculosis is defined as a positive culture for *Mycobacterium tuberculosis.* The RVCT is a national TB surveillance data reporting system in which all jurisdictions (the USA, US territories, and US-affiliated Pacific Islands) report all laboratory-verified TB cases.

## Results

A total of 2437 cases of TB were reported between 2010 and 2019. There was approximately a 14% positive rate among the three counties for HIV. In contrast, 47% of the individuals with active TB in all three counties were also diagnosed with diabetes mellitus. An average of 25% of active TB cases in these counties had a concurrent immunosuppressive condition other than HIV. Known contact with another active TB case was an identified risk factor in 18%, 17%, and 29% of reported TB cases in Dade, Broward, and Palm Beach counties, respectively.

The several relationships among the studied variables are described below.

Association between racial distribution and tuberculosis

**Figure 1:**
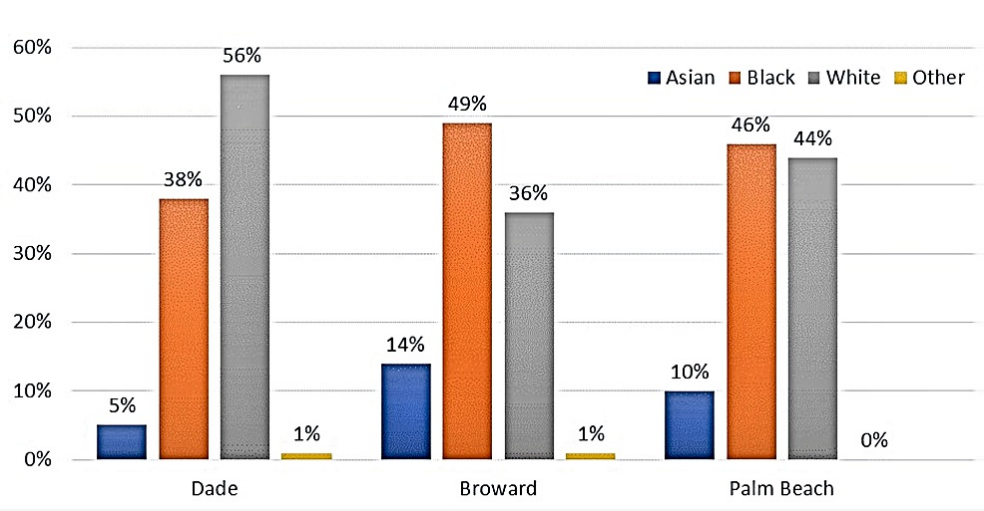
Reported cases by race for three metropolitan counties from 2010 to 2019 *Other: American Indian, Alaska Native, and Pacific Islander

**Figure 2:**
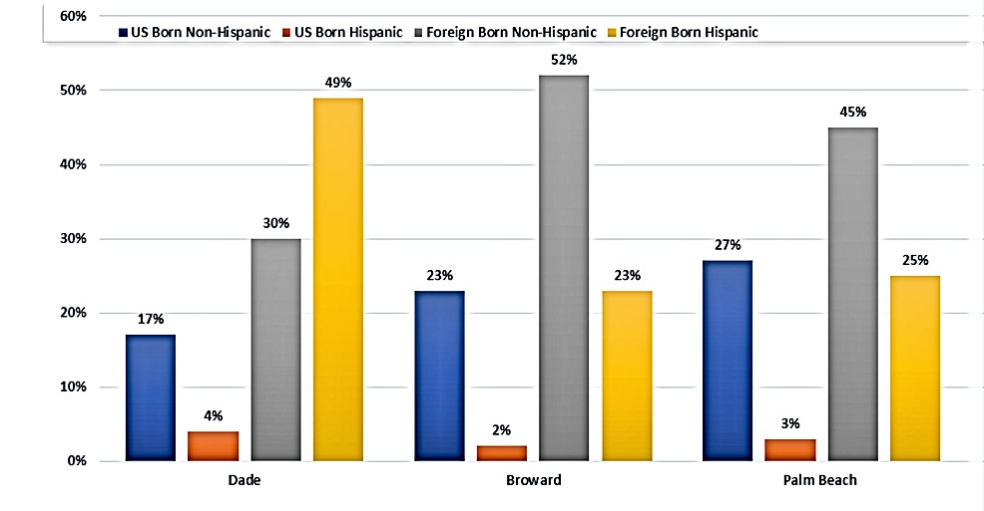
Reported cases by ethnicity for three metropolitan counties from 2010 to 2019

The categories of races used at the time of data collection were Asian, Black, White, and Other, which included American Indians, Alaska Natives, and Pacific Islanders (Figure 1). The reported TB cases were identified by ethnicity as US-born non-Hispanics, US-born Hispanics, foreign-born non-Hispanics, and foreign-born Hispanics (Figure 2).

In Dade County, 56% (n = 713) of TB cases diagnosed between 2010 and 2019 occurred in White individuals, and 49% of these cases were attributed to foreign-born Hispanic individuals (Figure 2). Roughly 38% (n = 492) of reported TB cases in Dade County were identified as Blacks/African Americans; portions of this TB-positive, Black/African American group are represented as the second-highest group within the foreign-born Hispanic and foreign-born non-Hispanic groups. Approximately 5% (n = 71) of reported TB cases in Dade County were attributed to individuals from the Asian race. The remaining races (American Indian, Alaskan Native, and Pacific Islander) accounted for 1% (n = 7) of reported cases. When reviewing the data for ethnicity, the US-born non-Hispanic category comprised 17% (n = 216) of the total reported TB cases, followed by 4% (n = 58) of the total cases reported in US-born Hispanic individuals (Figure 2).

Broward County slightly differed in reported TB cases by race and ethnicity (Figures 1, 2). Reported cases of TB affecting Black/African American individuals accounted for 49% (n = 327) of total cases and 52% (n = 349) of cases within foreign-born non-Hispanic individuals (Figures 1, 2). The remaining 36% (n = 242) of the total TB cases occurred in White individuals, followed by 14% (n = 91) of cases in Asian individuals.

Data from Palm Beach County had both Whites and Blacks demonstrating the bulk of the cases with 44% (n = 212) and 46% (n = 225), respectively (Figure 1); 10% (n = 47) of the cases affected Asians. Regarding ethnicity, 25% (n = 124) and 27% (n = 130) of the cases were foreign-born Hispanics and US-born non-Hispanics (Figure 2), respectively. Overall, the cases that were deemed foreign born were from the Caribbean and South America.

Association between the prevalence of HIV and tuberculosis

**Figure 3:**
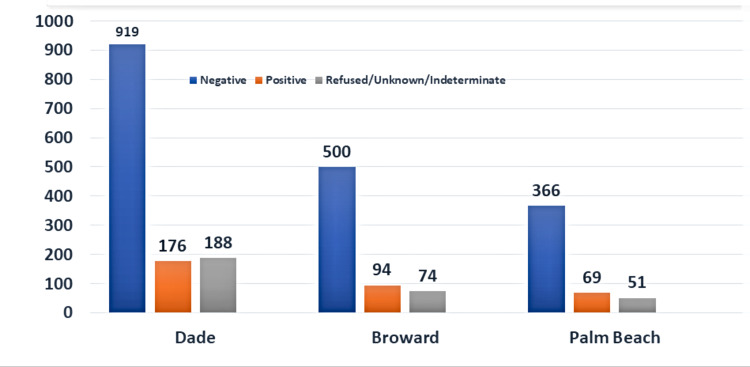
HIV status among TB cases in three metropolitan counties from 2010 to 2019

The HIV status of the TB cases in Dade, Broward, and Palm Beach counties reported from 2010 to 2019 were analyzed. In comparing the counties, approximately 70%-75% of the cases were negative for HIV, with active TB strongly linked to HIV disease (Table 1, Figure 3). There was approximately a 14% positive rate among the three counties for HIV.

**Table 1:** HIV status among TB cases in three metropolitan counties from 2010 to 2019

	Dade	Broward	Palm Beach
Negative	919 (71.6%)	500 (74.9%)	366 (69.1%)
Not Offered	59 (4.5%)	40 (6.0%)	11 (2.3%)
Positive	176 (13.7%)	94 (14.0%)	69 (14.2%)
Refused	129 (10%)	32 (4.8%)	32 (6.6%)
Indeterminate/Unknown	0	2 (0.3%)	8 (1.6%)
Total	1283	668	486

Association between the prevalence of HIV and others (diabetes, race, recent contact with active TB cases, non-HIV/AIDS immunosuppression, post-organ transplantation, and end-stage renal disease)

**Figure 4:**
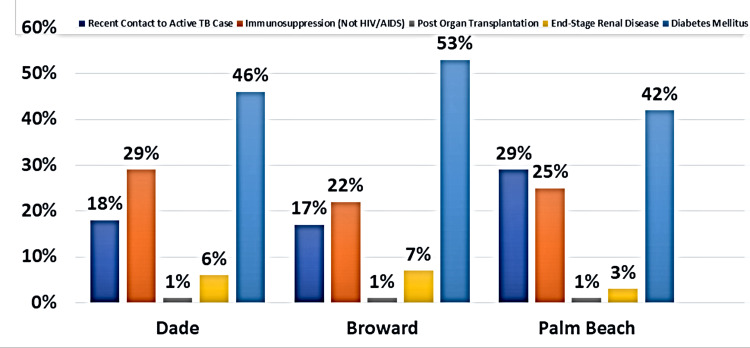
Other risk factors of active TB cases in three metropolitan counties from 2010 to 2019

The prevalence of diabetes is as follows: 46% in Dade, 53% in Broward, and 42% in Palm Beach (Figure 4). Comorbidity with an immunosuppressive state, such as an autoimmune condition, occurred in 29%, 22%, and 25% of reported TB cases in Dade, Broward, and Palm Beach counties, respectively (Figure 4). Another significant risk factor identified in the reported TB cases was contacts with another active TB case, as shown in 18% of cases in Dade County, 17% of cases in Broward County, and 29% of cases in Palm Beach County.

**Figure 5:**
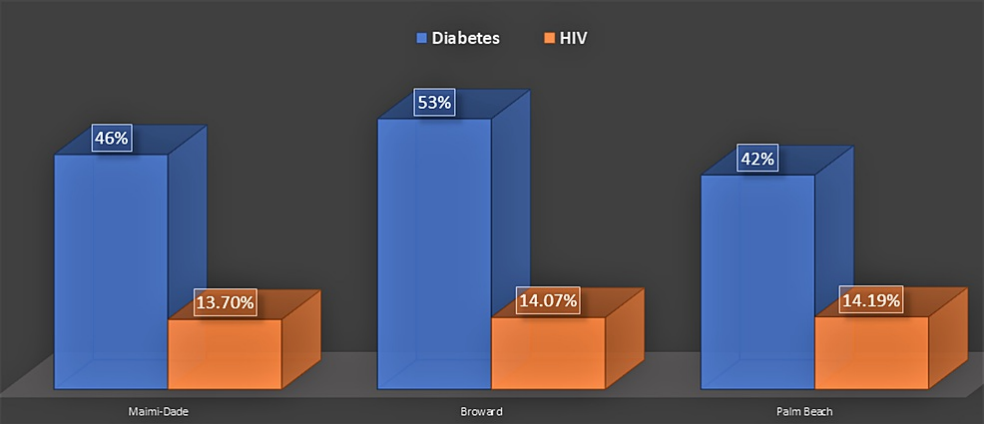
Prevalence of HIV and diabetes among TB cases in the three counties

Furthermore, the prevalence of diabetes and HIV in the sampled population was also analyzed (Figure 5).

## Discussion

The information included in the RVCT’s clinical summary report from Dade, Broward, and Palm Beach counties from 2010 to 2019 was reviewed and used in this study. The listing of the various types of tuberculosis cases for each year and previously identified risk factors that may have contributed to the development of active TB were noted. In addition, an assessment of the patient population was performed using the demographics section of the RVCT.

Each county was analyzed individually for specific risk factors, such as HIV, diabetes, and other potential causes that could have fostered the activation of TB. Moreover, details regarding the race and/or ethnicity of TB cases were also examined. The data was summarized for a nine-year period for each county. Microsoft Excel programming was used to perform computations and graphs for visual observation of data for possible correlations.

The regions were assessed based on race, ethnicity, and risk factors that could have contributed to the development of active tuberculosis. Initially, it was hypothesized that the risk factor of positive HIV status would have contributed significantly to the development of active tuberculosis. Based on the results, perhaps that initial assumption may not be accurate in these areas (Figure 5). When examining reported tuberculosis cases by race and ethnicity, foreign-born Hispanic individuals and those classified as White under race seem to experience the highest prevalence of TB in Dade County. The country of origin for most of these affected individuals is Haiti and Cuba. In the studied population, Haitian-born individuals were among the highest foreign-born individuals to develop active tuberculosis. One common factor associated with all three counties is the occurrence of active tuberculosis among Haitian-born individuals. Understanding the health beliefs and practices of this ethnic minority may be imperative to reducing TB disease.

Examining insight into the risk factors within the Haitian diaspora that could potentiate the activation of the disease from its latent state is paramount. Existing literature documents an increased prevalence of type 2 diabetes among Haitian immigrants [12]. The data from this review demonstrate that diabetes is the leading risk factor for TB disease in all three counties (Figure 4). Could diabetes be the elusive risk factor that is precluding TB disease suppression? Mirroring the same preventive strategy (active surveillance and treatment) used in HIV-positive patients to reduce TB could prove beneficial in individuals with uncontrolled and/or newly diagnosed diabetes.

Recent contact with another individual with active TB was the second most common risk factor in Palm Beach County after diabetes mellitus (Figure 4). In Dade and Broward counties, other immunosuppressive conditions (not HIV/AIDS) were deemed the second most common risk factor for developing active TB disease (Figure 4). The HIV status among TB cases in the three metropolitan counties was predominately negative. Knowledge of these facts guides providers, researchers, and public health professionals regarding community health promotion and preventive strategies. Understanding the specifics surrounding the development of active TB in patients with diabetes is crucial to defining an appropriate screening period. For instance, the optimal timing of TB screening via tuberculin skin test (TST) or interferon gamma release assay (IGRA) in newly diagnosed or uncontrolled diabetics should be determined. Undoubtedly, further research is necessary to unequivocally establish if preventive management, such as prophylactic treatment of LTBI, would be beneficial to reducing TB disease development in diabetics.

Study limitation

The limitations of this study are that the data obtained was minimal. There was no control group used. Extending the study to other counties would increase the generalizability of the findings and assist in the identification of trends regarding risk factors. There could be confounders that may have played a role in the trends seen in the analyzed sample size.

## Conclusions

Initially, this research set out to see the relationship of risk factors in the sampled population. Several trends were observed. The initial thought that HIV disease is the main culprit to TB activation may not be the case in the studied three counties in South Florida. The observed relationship showed otherwise in the results presented from the studied counties. With the observed higher cases of diabetes among the studied population, encouraging compliance and promoting LTBI management in newly diagnosed and uncontrolled diabetics may establish the next phase of TB prevention and control in this sampled region. A prospective cohort study is warranted to determine if a direct correlation exists between TB disease and diabetes and if LTBI management precludes the development of TB disease among this group. Increased understanding of risk factors, disease development, and the interactions between risk factors allows for implementing more effective and efficient prevention and screening programs.
